# Spatiotemporal APLNR Expression Dynamics During Oligodendroglial Remodeling of the Corpus Callosum in the Cuprizone Model

**DOI:** 10.3390/ijms27104519

**Published:** 2026-05-18

**Authors:** Lyubomir Gaydarski, Kristina Petrova, Nikola Stamenov, Alexandar Iliev, Stancho Stanchev, Pavel Rashev, Despina Pupaki, Milena Mourdjeva, Ivanka Kostadinova, Boycho Landzhov

**Affiliations:** 1 Department of Anatomy, Histology and Embryology, Medical University of Sofia, 1431 Sofia, Bulgaria; kristinapetrova270@gmail.com (K.P.); nikola.stamenov1@gmail.com (N.S.); dralexiliev@abv.bg (A.I.); stanchev_1989@abv.bg (S.S.); landzhov_medac@abv.bg (B.L.); 2 Institute of Biology and Immunology of Reproduction, Bulgarian Academy of Sciences, 1113 Sofia, Bulgaria; pavel_rashev@abv.bg (P.R.); poupaki_desi@abv.bg (D.P.); milena_mourdjeva@abv.bg (M.M.); 3 Department of Pharmacology, Pharmacotherapy and Toxicology, Medical University of Sofia, 1000 Sofia, Bulgaria; i.kostadinova@pharmfac.mu-sofia.bg

**Keywords:** apelinergic system, white matter remodulation, oligodendrocyte progenitor cell (OPC) dynamics, experimental model of MS, neurodegeneration

## Abstract

Demyelinating disorders such as multiple sclerosis are characterized by oligodendrocyte loss and insufficient remyelination. The cuprizone model provides a well-established experimental system for studying these processes. The apelinergic system, including the apelin receptor (APLNR), has been implicated in neuroprotection and central nervous system homeostasis. However, its role in white matter demyelination and repair remains incompletely understood. This study aimed to characterize the spatial and temporal dynamics of APLNR expression in relation to oligodendrocyte lineage cells in the corpus callosum (CC) during demyelination and remyelination. Demyelination was induced in 8-week-old C57BL/6 mice by 0.2% cuprizone supplementation in their drinking water for 5 weeks, followed by 5 weeks remyelination phase after toxin withdrawal. Histological assessment using Luxol Fast Blue/Cresyl violet staining was performed to evaluate structural changes in the CC. Immunohistochemistry and confocal microscopy were used to analyze APLNR expression, GST-π^+^ cells, and NG2^+^ cells, including their spatial distribution and co-localization. Quantitative analyses and correlation tests were conducted to assess relationships between cellular markers and CC area. Demyelination resulted in significant reduction in CC area and a marked decrease in GST-π^+^ cells, accompanied by a robust increase in NG2^+^ cells, while remyelination led to partial structural and cellular recovery. APLNR expression increased progressively from control to demyelination and further during remyelination, exhibiting pronounced regional heterogeneity with higher levels in lateral CC regions. Confocal analysis demonstrated increasing co-localization of APLNR with NG2^+^ cells, particularly during remyelination. Correlation analyses identified GST-π^+^ cell density as the strongest predictor of CC area, whereas APLNR showed phase-dependent associations, including a positive correlation with GST-π^+^ cells during remyelination and a negative relationship with NG2^+^ cells during demyelination. APLNR expression is dynamically regulated during cuprizone-induced demyelination and remyelination and is closely associated with oligodendrocyte lineage cell responses. Its increased expression and enhanced co-localization with NG2^+^ cells during remyelination suggest a potential role in endogenous repair processes. However, as the findings are based on descriptive analyses, further functional studies are required to determine the mechanistic contribution of APLNR signaling and its potential as a therapeutic target in demyelinating diseases.

## 1. Introduction

A leading cause of demyelination is multiple sclerosis (MS), a chronic neurodegenerative autoimmune disease [1]. Its pathogenesis involves the formation of focal lesions in the white matter of the brain and spinal cord [2]. The principal neuropathological features of MS include inflammation, demyelination, axonal injury, astrogliosis, and the loss of oligodendrocytes [2]. Inflammatory infiltration of T and B lymphocytes, macrophages, and soluble mediators into perivascular and parenchymal regions drives demyelination by promoting the destruction of oligodendrocyte-derived myelin. The resulting disruption of saltatory conduction leads to the characteristic neurological deficits of the disease [3].

To investigate the mechanisms of demyelination and remyelination, several experimental models have been developed. Among them, the cuprizone (CPZ)-induced demyelination model reproduces many of the histopathological features observed in MS patients [4]. Cuprizone, a copper-chelating compound, induces mitochondrial dysfunction by increasing superoxide anion production and reducing the activities of copper–zinc superoxide dismutase and cytochrome C oxidase [5,6,7]. This results in rapid demyelination, gliosis, and apoptosis of oligodendrocytes [5]. The cuprizone model is particularly valuable for studying remyelination, as cessation of the agent leads to spontaneous proliferation of oligodendrocyte precursors and the restoration of myelin [8].

In contrast, the myelin oligodendrocyte glycoprotein (MOG)-induced Experimental Autoimmune Encephalomyelitis (EAE) model is more suitable for exploring the autoimmune aspects of MS [9]. MOG, a myelin sheath protein from the immunoglobulin superfamily, can be recognized as an autoantigen by the immune system, provoking inflammation and demyelination in the CNS [9]. In mice, this immune response produces a relapsing–remitting course of disease, progressive motor dysfunction, and various neurological symptoms [10]. Other experimental systems include the lysolecithin (lysophosphatidylcholine, LPC) and Theiler’s Murine Encephalomyelitis Virus (TMEV) models [11]. While the lysolecithin model induces focal, well-defined demyelinated lesions [12], and the EAE model reproduces immune-mediated mechanisms of MS and has been instrumental in developing therapies for relapsing–remitting disease [13], both have limitations. The EAE model’s reliance on CD4^+^ T-cell-mediated inflammation, its artificial induction, and limited representation of progressive pathology reduce its utility for studying neurodegeneration [14].

In this regard, the cuprizone model provides a more controlled system by eliminating autoimmune components and generating clearly defined phases of demyelination and remyelination [15,16]. It induces consistent, time-dependent demyelination primarily restricted to the CNS, avoiding confounding effects from peripheral inflammation [15,16,17]. The corpus callosum (CC) is particularly vulnerable to cuprizone toxicity and is commonly used as the primary region for assessing myelin loss and repair [11,18,19,20]. The C57BL/6 mouse strain is most frequently employed for this model, as it shows pronounced myelin and oligodendrocyte loss compared to other strains such as CD1 [21,22].

The apelinergic system, comprising the apelin receptor (APLNR) and its endogenous ligands apelin and elabela, plays multiple physiological roles [23]. Apelin exerts pleiotropic effects across cardiovascular, renal, immune, and neuroendocrine systems, as well as in maintaining homeostasis and modulating neurodegenerative processes [24,25,26,27]. Importantly, the apelinergic system has demonstrated neuroprotective properties, including the modulation of inflammation, prevention of cell death, and reduction in oxidative stress [28]. Recent studies have examined the role of the apelin/APLNR axis in demyelination and remyelination within the subventricular zone (SVZ) [29]. The present study aims to extend these findings by evaluating its role in the CC—one of the main targets of cuprizone-induced demyelination [29].

Glutathione S-transferases (GSTs) are a family of detoxifying enzymes with eight subclasses (alpha(α), mu(µ), pi(π), theta(θ), sigma(σ), kappa(κ), zeta(ζ), and omega(ω)) that catalyze the conjugation of glutathione to a broad range of substrates [30,31]. The GST-π isoform is expressed in neurons and glial cells and is localized both in the cytoplasm of mature oligodendrocytes and in the cytoplasm and nuclei of glial cells in the human brain [31]. Tamura et al. identified two GST-π subtypes in the cerebral cortex—C-type (cytoplasmic, primarily in mature oligodendrocytes) and N-type (nuclear, mainly in NG2^+^ progenitor cells)—and proposed that proliferating N-type cells differentiate stepwise into C-type oligodendrocytes [31]. Supporting this, a previous study has reported alterations in GST-π expression and NG2^+^ cell dynamics during cuprizone-induced demyelination, suggesting disrupted oligodendrocyte homeostasis and activation of progenitor-mediated repair responses [32].

NG2^+^ cells, also known as OPCs, are a distinct population of glial progenitors that can differentiate not only into oligodendrocytes but also, under certain conditions, into astrocytes [33,34]. They form both excitatory and inhibitory synaptic contacts with neurons, highlighting their active involvement in CNS signaling networks [35]. During cuprizone-induced demyelination, NG2^+^ glial cells exhibit dynamic changes. Some studies have reported an increase in NG2^+^ cell density in CC during cuprizone-induced demyelination, suggesting a proliferative response to oligodendrocyte loss [36], whereas others have observed a transient reduction in NG2^+^ cells in SVZ during the demyelination phase followed by recovery during remyelination [29].

By examining the dynamics of APLNR signaling, NG2^+^ progenitor cell behavior, and GST-π expression, this study aims to elucidate mechanisms underlying oligodendrocyte loss and repair during cuprizone-induced demyelination in C57BL/6 mice.

## 2. Results

### 2.1. Luxol Fast Blue (LFB)/Cresyl Violet Histological Assessment of CC

**Figure 1:**
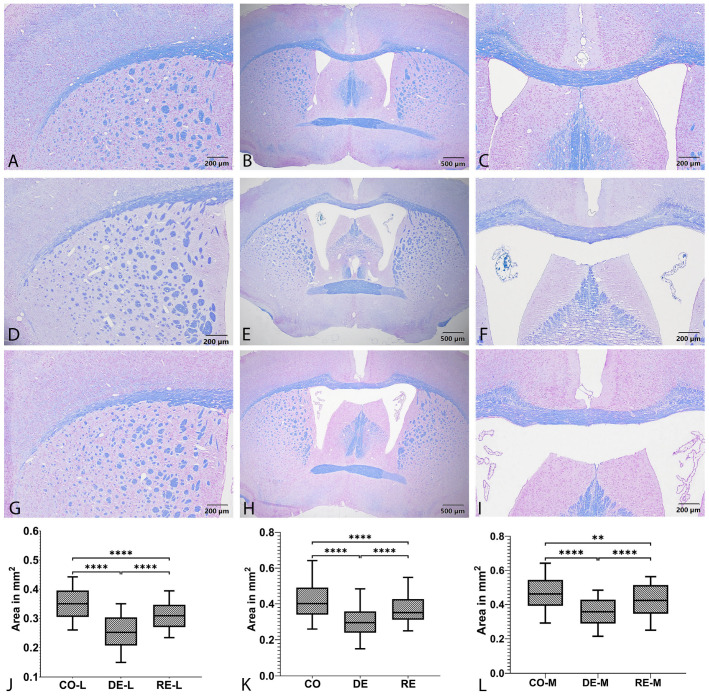
Histological assessment of the CC on coronal brain sections stained with Luxol Fast Blue and Cresyl violet in the CO (**A**–**C**), DE (**D**–**F**), and RE (**G**–**I**) groups. (**B**,**E**,**H**) Overview images of the whole CC; scale bar = 500 µm. (**A**,**D**,**G**) Overview images of the lateral CC; scale bar = 200 µm. (**C**,**F**,**I**) Overview images of the medial CC; scale bar = 200 µm. Graphical representation of the CC area (mm^2^) in the CO, DE, and RE groups, presented as box and whisker plots. (**J**)—whole CC area; (**K**)—area in the CC-L; (**L**)—area in the CC-M. Statistical significance: **** *p* < 0.0001; ** *p* < 0.01.

For histological assessment of demyelination and remyelination, the CC area was measured on coronal sections stained with LFB/Cresyl violet (Figure 1A–J). In the DE group, pale-stained regions associated with myelin loss were evident (Figure 1D–F). A consistent group effect was observed for CC area, with the CO group exhibiting the highest mean total CC area (0.421 mm^2^), followed by the RE group (0.367 mm^2^), whereas the DE group showed the lowest values (0.324 mm^2^). In the subgroup analysis, the medial CC was larger than the lateral CC in all groups: CO-M, 0.466 mm^2^ versus CO-L, 0.351 mm^2^; RE-M, 0.423 mm^2^ versus RE-L, 0.310 mm^2^; and DE-M, 0.356 mm^2^ versus DE-L, 0.254 mm^2^. The greatest relative reduction was observed in the lateral CC of the DE group, suggesting that demyelination affected the lateral regions more prominently. Kruskal–Wallis analysis revealed significant differences in total CC area among groups (*p* < 0.0001). Dunn’s post hoc test showed significant differences between CO and DE (*p* < 0.0001), DE and RE (*p* < 0.0001), and CO and RE (*p* < 0.0001) (Figure 1J). For the lateral CC (CC-L), the Kruskal–Wallis test was also significant (*p* < 0.0001), and all pairwise comparisons were significant (CO-L vs. DE-L, CO-L vs. RE-L, and DE-L vs. RE-L; *p* < 0.0001 for all) (Figure 1K). For the medial CC (CC-M), the Kruskal–Wallis test remained significant (*p* < 0.0001); post hoc analysis showed significant differences between CO-M and DE-M and between DE-M and RE-M (*p* < 0.0001 for both), while the difference between CO-M and RE-M was also significant, albeit at a lower level (*p* < 0.01) (Figure 1L).

### 2.2. Immunohistochemical Assessment of CC

#### 2.2.1. APLNR Immunoreactivity

**Figure 2:**
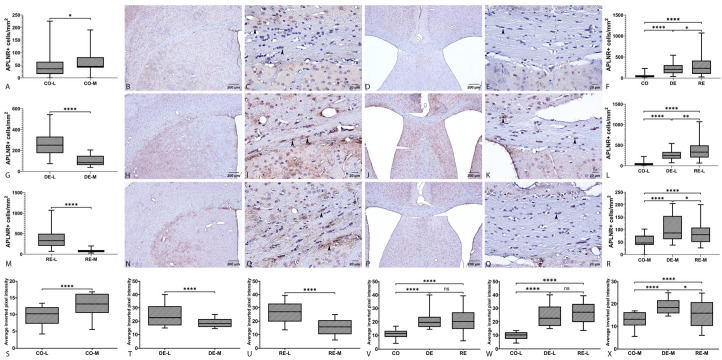
APLNR immunohistochemistry in the corpus callosum (CC) during demyelination and remyelination. Lateral corpus callosum (CC-L); Medial corpus callosum (CC-M). (**A**) Box-and-whisker plot showing APLNR^+^ cell density in CC-L versus CC-M in control animals (CO). (**B**–**E**) Representative immunohistochemical images of APLNR staining in the CC: (**B**) low-magnification overview and (**C**) higher magnification view of the CC-L in CO; (**D**) low-magnification overview and (**E**) higher magnification view in the demyelination group (DE). (**F**) Box-and-whisker plot comparing APLNR^+^ cell density among CO, DE and remyelination (RE) groups. (**G**) Box-and-whisker plot comparing APLNR^+^ cell density between CC-L and CC-M in the DE group. (**H**–**K**) Representative APLNR immunostaining during demyelination: (**H**) low-magnification overview of the CC-L and (**I**) higher magnification of the CC-L; (**J**) low-magnification overview of the CC-M and (**K**) higher magnification view of the CC-M in DE (**L**). Box-and-whisker plot comparing APLNR^+^ cell density among CO-L, DE-L and RE-L. (**M**) Box-and-whisker plot comparing APLNR^+^ cell density between CC-L and CC-M in the RE group. (**N**–**Q**) Representative APLNR immunostaining during remyelination: (**N**) low-magnification overview of the CC-L and (**O**) higher magnification of CC-L; (**P**) low-magnification overview of CC-L and (**Q**) higher magnification view of CC-M in RE. Arrow heads indicate immunopositive cells. (**R**) Box-and-whisker plot comparing APLNR^+^ cell density among CO-M, DE-M and RE-M. (**S**–**U**) Box-and-whisker plots showing the mean inverted pixel intensity (index of APLNR staining intensity) comparing CC-L and CC-M in CO (**S**), DE (**T**) and RE (**U**) groups. (**V**–**X**) Box-and-whisker plots comparing mean inverted pixel intensity among experimental groups in the whole CC (**V**), CC-L (**W**) and CC-M (**X**). Scale bars: 200 µm (low magnification) and 20 µm (high magnification). Box plots represent median with interquartile range and minimum–maximum values. Statistical annotations: **** *p* < 0.0001; ** *p* < 0.01; * *p* < 0.05; ns, not significant.

APLNR immunoreactivity in the CC was predominantly cytoplasmic and displayed a distinct spatial organization that varied with subregion (medial versus lateral) and experimental condition (control, demyelination, remyelination). In control animals, APLNR signal was localized mainly to the cytoplasm of individual cells found both in the subependymal region and in the central zone of the CC-M. In the CC-L regions the signal was more diffuse, and positive cells were relatively scattered (Figure 2B–E). During demyelination, APLNR expression intensity increased and its spatial distribution changed. In the CC-L there was both intracellular labeling (cytoplasmic and membranous) clearly delineating individual cells and a pronounced extracellular APLNR signal that was not associated with adjacent cell nuclei (Figure 2H,I). This extracellular pattern could reflect labeling of axonal bundles or of glial processes (astrocytic or microglial). In the CC-M, a mixed pattern was observed, with an increased cellular component together with moderate fibrillar staining; in some fields, intense perivascular APLNR^+^ cells were evident (Figure 1J,K). During remyelination, subcellular localization shifted further. In CC-L, the APLNR signal more frequently exhibited both cellular and extracellular, network-like components that were more evenly distributed than during demyelination (Figure 2N,O). In the CC-M of remyelinating animals, APLNR expression was predominantly cellular (Figure 2P,Q).

The mean number of APLNR^+^ cells per field (mean ± SD) was 2.85 ± 2.98 in the control group, 11.71 ± 6.17 in the demyelination group, and 16.75 ± 10.65 in the remyelination group, corresponding to approximate densities of 53.46 cells/mm^2^ (CO), 218.35 cells/mm^2^ (DE), and 291.20 cells/mm^2^ (RE). The Kruskal–Wallis test revealed a significant effect of group on the number of APLNR^+^ cells (*p* < 0.0001). Dunn’s multiple comparison post hoc test demonstrated statistically significant differences for all pairwise comparisons (CO vs. DE and CO vs. RE, *p* < 0.0001; DE vs. RE, *p* = 0.002), indicating a clear stepwise increase in cell density across groups (CO < DE < RE). These results indicate a low basal density of APLNR^+^ cells in the control group, a marked increase during demyelination, and a further increase during remyelination (Figure 1F). In the CC-L, the mean number of APLNR^+^ cells per field was 1.92 ± 1.90 (density 49.14 cells/mm^2^) in CO-L, 12.57 ± 6.54 (density 259.74 cells/mm^2^) in DE-L, and 21.08 ± 10.66 (density 372.07 cells/mm^2^) in RE-L. The Kruskal–Wallis test for the lateral region was highly significant (*p* < 0.0001). Dunn’s post hoc analysis demonstrated statistically significant differences between all pairwise comparisons (CO-L vs. DE-L and CO-L vs. RE-L, *p* < 0.0001; DE-L vs. RE-L, *p* < 0.01), confirming a clear stepwise increase in densities (CO-L < DE-L < RE-L). In the CC-M, the mean number of APLNR^+^ cells per field was 4.41 ± 3.58 (density 62.12 cells/mm^2^) in CO-M, 9.39 ± 4.09 (density 106.97 cells/mm^2^) in DE-M, and 7.01 ± 4.15 (density 88.37 cells/mm^2^) in RE-M. The Kruskal–Wallis test revealed a significant group effect (*p* < 0.0001). Dunn’s post hoc comparisons showed significant differences for all pairwise comparisons (CO-M vs. DE-M and CO-M vs. RE-M, *p* < 0.0001; DE-M vs. RE-M, *p* < 0.05). Thus, in the medial segment, the density of APLNR^+^ cells increased from control to demyelination, followed by a partial reduction during remyelination (Figure 2R). Within-group comparisons using the two-tailed Mann–Whitney U test revealed a consistent lateral–medial heterogeneity across all experimental groups. In all cases, the differences between the lateral and medial compartments were statistically significant (CO-L vs. CO-M, *p* < 0.05; DE-L vs. DE-M and RE-L vs. RE-M, *p* < 0.001) (Figure 2A,G,M). A similar pattern was observed in the analysis of APLNR expression intensity in the CC. The Kruskal–Wallis test demonstrated a significant difference among groups (*p* < 0.001). Post hoc comparisons showed that the control group differed significantly from both the demyelination and remyelination groups (CO vs. DE and CO vs. RE, *p* < 0.001), whereas no statistically significant difference was observed between DE and RE (Figure 2V). Analysis of APLNR expression in the CC-L also revealed significant group differences (Kruskal–Wallis, *p* < 0.001). Post hoc comparisons indicated that CO-L differed significantly from both DE-L and RE-L (*p* < 0.0001 for each), while no significant difference was detected between DE-L and RE-L (*p* > 0.05) (Figure 2W). In the CC-M, Kruskal–Wallis analysis showed significant intergroup differences (*p* < 0.0001), and all subsequent post hoc comparisons were statistically significant (*p* < 0.0001) (Figure 2X). Finally, within-group comparisons of APLNR expression levels between the lateral and medial subregions, performed using a two-tailed Mann–Whitney U test, revealed a consistent lateral–medial heterogeneity across all experimental groups. In each case, statistically significant differences were observed between the lateral and medial compartments (*p* < 0.0001), indicating a stable regional difference in APLNR expression regardless of the experimental condition (Figure 2S–U).

#### 2.2.2. GST-π Immunoreactivity

**Figure 3:**
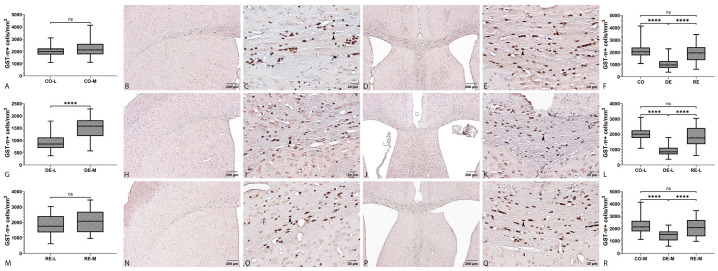
GST-π immunohistochemistry in the corpus callosum (CC) during control, demyelination and remyelination. (**A**–**F**) Control (CO). (**A**) Box-and-whisker plot comparing GST-π^+^ cell density (cells/mm^2^) in CC-L versus CC-M in CO animals. (**B**) Low-magnification overview of the lateral CC (CC-L). (**C**) High-magnification view of the CC-L. (**D**) Low-magnification overview of the medial CC (CC-M). (**E**) High-magnification view of the CC-M. (**F**) Box-and-whisker plot comparing GST-π^+^ cell density among CO, DE and RE groups for the CC (CO, DE, RE). (**G**–**L**) Demyelination (DE). (**G**) Box-and-whisker plot comparing GST-π^+^ cell density in CC-L versus CC-M in DE. (**H**) Low-magnification overview of the CC-L during demyelination. (**I**) High-magnification view of CC-L in DE. (**J**) Low-magnification overview of the CC-M during demyelination. (**K**) High-magnification view of CC-M in DE. (**L**) Box-and-whisker plot comparing GST-π^+^ cell density across groups for the CC-L (CO-L, DE-L, RE-L). (**M**–**R**) Remyelination (RE). (**M**) Box-and-whisker plot comparing GST-π^+^ cell density in CC-L versus CC-M in RE animals. (**N**) Low-magnification overview of the CC-L during remyelination. (**O**) High-magnification view of CC-L in RE. (**P**) Low-magnification overview of the CC-M. (**Q**) High-power view of CC-M in RE. Arrow heads indicate immunopositive cells. (**R**) Box-and-whisker plot comparing GST-π^+^ cell density among CO-M, DE-M and RE-M. CC-L, lateral corpus callosum; CC-M, medial corpus callosum. Scale bars: low = 200 µm; high = 20 µm. All box plots display median, interquartile range and minimum–maximum values. Statistical comparisons are shown on the graphs; significance markers: **** *p* < 0.0001, ns = not significant.

GST-π immunoreactivity in the CC was localized predominantly in the cytoplasm of spherical cells morphologically consistent with mature oligodendrocytes. In control animals, GST-π staining showed a homogeneous spatial distribution throughout the coronal section of the CC. Individual GST-π^+^ cells appeared as well-defined spheroidal profiles with intense cytoplasmic labeling and minimal fibrillar staining (Figure 3B–E). After 5 weeks of cuprizone administration, the overall spatial pattern of GST-π expression was preserved; however, a clear focal reduction in the density of GST-π^+^ cells was observed in the CC-L (Figure 3H,I). These focal deficits appeared as local areas containing fewer spheroid cells and weaker cytoplasmic staining compared with adjacent regions. In the CC-M, the reduction in GST-π^+^ cells was more moderate and was more often expressed as a diffuse decrease in staining intensity rather than complete loss (Figure 3J,K). Consequently, the medial region retained a relatively more stable population of GST-π^+^ cells compared with the lateral zones. This pattern suggests that lateral oligodendrocyte populations may be more susceptible to cuprizone-induced stress than medial populations. During the remyelination phase, a partial recovery in the density of GST-π^+^ cells was observed. In the lateral regions that exhibited reductions during demyelination, an increased number of GST-π^+^ cells and restoration of cytoplasmic staining intensity were detected, although in some animals levels did not fully return to those observed in controls (Figure 3N,O). In the CC-M, the spatial distribution of GST-π regained a more homogeneous appearance similar to that seen in the control group (Figure 3P,Q). The observed partial recovery during remyelination supports the hypothesis of dynamic remodeling of mature oligodendrocytes and/or differentiation of oligodendrocyte precursor cells following withdrawal of the demyelinating agent. GST-π exhibited a pattern opposite to that observed for APLNR. The control group showed the highest cell densities, the demyelination group demonstrated a pronounced and significant reduction, and the remyelination group showed partial recovery toward control levels. The measured densities were 2173.91 cells/mm^2^ in the control group, 1277.89 cells/mm^2^ in the demyelination group, and 2104.89 cells/mm^2^ in the remyelination group. The Kruskal–Wallis test revealed significant differences among groups (*p* < 0.0001). Post hoc comparisons showed significant differences between the control and demyelination groups (*p* < 0.0001) as well as between the demyelination and remyelination groups (*p* < 0.0001), whereas no statistically significant difference was detected between the control and remyelination groups (*p* > 0.05). These findings indicate that demyelination markedly reduces GST-π expression, whereas remyelination largely restores expression to levels comparable to those in control animals (Figure 3F). Subregional analyses followed the same general pattern (CO > RE > DE). In the CC-L the mean densities were 2013 cells/mm^2^ in the control group, 908 cells/mm^2^ in the demyelination group, and 1883 cells/mm^2^ in the remyelination group. The Kruskal–Wallis test was highly significant (*p* < 0.0001). Pairwise comparisons showed significant differences between CO-L and DE-L (*p* < 0.0001) and between DE-L and RE-L (*p* < 0.0001), whereas the comparison between CO-L and RE-L did not reach statistical significance (*p* > 0.05) (Figure 3L). A similar pattern was observed in the CC-M, where the mean cell densities were 2207 cells/mm^2^ in the control group, 1424 cells/mm^2^ in the demyelination group, and 2063 cells/mm^2^ in the remyelination group. The Kruskal–Wallis test demonstrated significant differences among groups (*p* < 0.0001). Post hoc analysis confirmed significant differences between CO-M and DE-M (*p* < 0.0001) and between DE-M and RE-M (*p* < 0.0001), while no significant difference was found between CO-M and RE-M (*p* > 0.05) (Figure 3R). Within-group regional comparisons revealed a significant difference only in the demyelination group, where GST-π^+^ cell density differed between the lateral and medial compartments (DE-L vs. DE-M, *p* < 0.0001). No significant regional differences were observed in the control or remyelination groups (CO-L vs. CO-M and RE-L vs. RE-M, *p* > 0.05; two-tailed Mann–Whitney U test) (Figure 3A,G,M).

#### 2.2.3. NG2 Immunoreactivity

**Figure 4:**
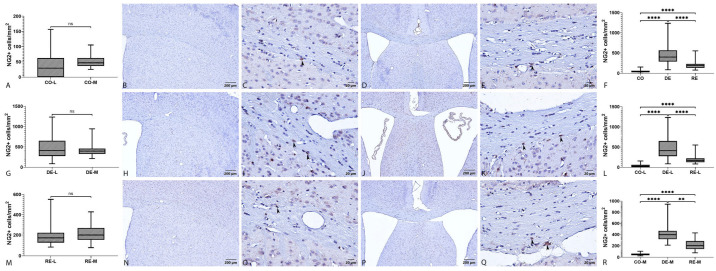
NG2 immunohistochemistry in the corpus callosum (CC) during control, demyelination and remyelination. Lateral corpus callosum (CC-L); Medial corpus callosum (CC-M). (**A**–**F**) Control (CO). (**A**) Box-and-whisker plot comparing NG2^+^ cell density (cells/mm^2^) in CC-L versus CC-M in CO. (**B**) Low-magnification overview of the CC-L. (**C**) High-magnification view of the CC-L. (**D**) Low-magnification overview of the CC-M. (**E**) High-magnification view of the CC-M. (**F**) Box-and-whisker plot comparing NG2^+^ cell density among CO, DE and RE groups for the CC (CO, DE, RE). (**G**–**L**) Demyelination (DE). (**G**) Box-and-whisker plot comparing NG2^+^ cell density in CC-L versus CC-M in DE. (**H**) Low-magnification overview of the CC-L during demyelination. (**I**) High-magnification view of CC-L in DE. (**J**) Low-magnification overview of the CC-M in DE. (**K**) High-magnification view of CC-M in DE. (**L**) Box-and-whisker plot comparing NG2^+^ cell density across groups for the CC-L (CO-L, DE-L, RE-L). (**M**–**R**) Remyelination (RE). (**M**) Box-and-whisker plot comparing NG2^+^ cell density in CC-L versus CC-M in RE. (**N**) Low-magnification overview of the CC-L during remyelination. (**O**) High-magnification view of CC-L in RE. (**P**) Low-magnification overview of the CC-M. (**Q**) High-power view of CC-M in RE. Arrow heads indicate immunopositive cells. (**R**) Box-and-whisker plot comparing NG2^+^ cell density among CO-M, DE-M and RE-M. Scale bars: low = 200 µm; high = 20 µm. All box plots display median, interquartile range and minimum–maximum values. Statistical comparisons are shown on the graphs; significance markers: **** *p* < 0.0001, ** *p* < 0.01, ns = not significant.

NG2 immunoreactivity was observed predominantly in the cytoplasm of individual, sparsely distributed cells characterized by well-defined cell bodies and multiple thin processes, consistent with the morphological features of OPCs. In control specimens, NG2^+^ cells showed a relatively uniform distribution throughout the examined CC. In the medial region, positive cells were primarily concentrated in the subependymal area and the central CC-M zone, whereas in the lateral region they were more dispersed and mainly localized within the central portion of the CC-L (Figure 4B–E). Induction of demyelination resulted in a noticeable reorganization of NG2^+^ cell populations. Small linear clusters appeared in several observed fields, typically consisting of three to four cell bodies. These clusters were localized subependymally and along the central portion of the CC-M, as well as within the central areas of the CC-L. Cluster formation was accompanied by morphological changes, with some cells exhibiting enlarged cell bodies and thicker, elongated processes (Figure 4H–K). During the remyelination phase, NG2^+^ populations displayed a shift toward a more dispersed pattern. Many of the small clusters regressed, and the overall cellular distribution approached the pattern observed in control specimens. In CC-M, individual NG2^+^ cells again occupied predominantly subependymal and central positions, whereas in the CC-L a relative homogeneity of NG2^+^ cell distribution was restored (Figure 4N–Q). Across the CC, the mean number of NG2^+^ cells per field was lowest in the control group (CO = 2.78; density 46.54 cells/mm^2^), markedly increased in the demyelination group (DE = 23.15; density 454.55 cells/mm^2^), and intermediate in the remyelination group (RE = 11.26; density 200.53 cells/mm^2^). The Kruskal–Wallis test revealed a strong overall group effect (*p* < 0.0001). Subsequent Dunn’s post hoc analysis demonstrated statistically significant differences between all group pairs (CO vs. DE, CO vs. RE, and DE vs. RE; *p* < 0.05). These results indicate a clear pattern of CO < RE < DE with respect to the number and density of NG2^+^ cells in the CC, consistent with activation and proliferation of NG2-expressing cells during demyelination and their partial reduction during remyelination (Figure 4F). In the CC-L, the mean number of NG2^+^ cells per field was also lowest in the control group (CO-L = 2.05; density 43.03 cells/mm^2^), markedly elevated in the demyelination group (DE-L = 20.21; density 468.26 cells/mm^2^), and intermediate in the remyelination group (RE-L = 9.07; density 190.56 cells/mm^2^). The Kruskal–Wallis test for the lateral subregion indicated a significant overall group effect (*p* < 0.0001). Dunn’s post hoc analysis revealed significant differences between all pairwise comparisons (CO-L vs. DE-L, CO-L vs. RE-L, and DE-L vs. RE-L; *p* < 0.0001). These results demonstrate a pronounced increase in NG2^+^ cells in the lateral CC during demyelination, followed by partial attenuation of this response during remyelination (Figure 4L). In the CC-M, the mean number of NG2^+^ cells per field was lowest in the control group (CO-M = 3.85; density 51.97 cells/mm^2^), substantially increased in the demyelination group (DE-M = 28.88; density 426.14 cells/mm^2^), and intermediate in the remyelination group (RE-M = 15.24; density 219.10 cells/mm^2^). Kruskal–Wallis analysis for the medial subregion revealed a strong group effect (*p* < 0.0001). All subsequent Dunn’s post hoc comparisons were statistically significant (CO-M vs. DE-M and CO-M vs. RE-M, *p* < 0.0001; DE-M vs. RE-M, *p* < 0.01). These findings demonstrate a pronounced increase in NG2^+^ cell number and density during demyelination, followed by partial reduction during remyelination, highlighting an active cellular response in the medial CC (Figure 4R). Within-group comparisons between lateral and medial subregions, performed using a two-tailed Mann–Whitney U test, did not reveal statistically significant differences under any of the three experimental conditions (CO, DE, or RE; *p* > 0.05). Despite this, noticeable variability in NG2^+^ cell distribution was observed, particularly in the lateral regions, reflected by larger deviations in cell counts. This variability corresponds to the morphological distribution of NG2^+^ cells and their tendency to form small cellular clusters (Figure 4A,G,M).

### 2.3. Confocal Microscopy

**Figure 5:**
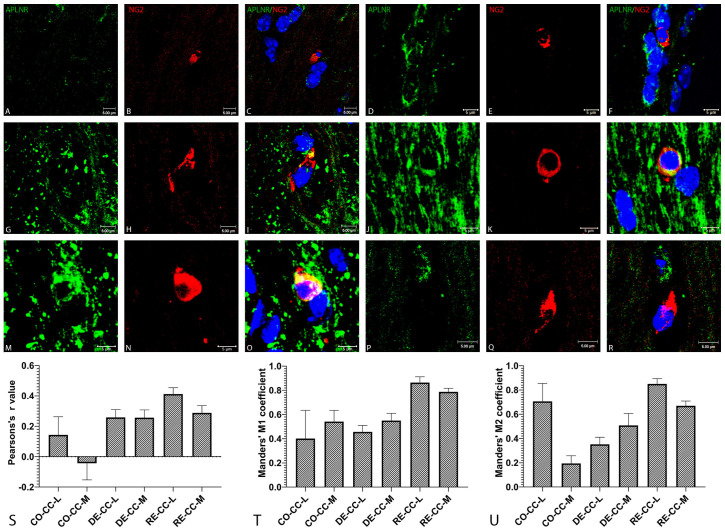
Confocal immunofluorescence of APLNR (green), NG2 (red), nuclei (Hoechst, blue) and colocalization between APLNR and NG2 (yellow) in the corpus callosum (CC). (**A**–**F**) Control (CO). Representative images are shown for CC-L (**A**–**C**) and CC-M (**D**–**F**) in CO. (**G**–**L**) Demyelination (DE); Representative images are shown for CC-L (**G**–**I**) and CC-M (**J**–**L**) in DE. (**M**–**R**) Remyelination (RE). Representative images are shown for CC-L (**M**–**O**) and CC-M (**P**–**R**). (**C**,**I**,**O**,**F**,**L**,**R**) Images display merged fluorescence channels (APLNR in green, NG2 in red) with nuclear counterstain (Hoechst, blue). Scale bar = 5 μm. (**S**)—graphical representation of the Pearson’s r value; (**T**)—graphical representation of Manders’ M1 coefficient; (**U**)—graphical representation of Manders’ M1 coefficient.

Confocal microscopy confirmed the observations obtained by immunohistochemistry and further clarified the subcellular localization of APLNR in relation to NG2^+^ cells in the CC. In the control group, the number of NG2^+^ cells was low and the APLNR signal was weak. Co-localization between the two markers was limited to occasional cells and appeared as partial overlap between channels without clear subcellular integration. No apparent morphological differences were observed between the lateral and medial regions of the CC; in both regions, NG2^+^ cells appeared relatively small with oval or round cell bodies. Induction of demyelination resulted in a marked increase in the density of NG2^+^ cells and an elevation in APLNR expression within the examined CC regions (Figure 5A–F). In the demyelination group, notable morphological differences were observed in NG2^+^ cells. In the CC-L, NG2^+^ cells frequently exhibited pyknotic nuclei, and the NG2 signal (red channel) appeared heterogeneous and fragmented, suggesting that some of the observed cells might be undergoing apoptosis. Interestingly, despite these alterations, some of these cells still showed partial APLNR expression, with co-localization typically restricted to one pole of the cell (Figure 5G–I). In contrast, NG2^+^ cells in the CC-M appeared as rounded cells with clear co-localization with APLNR in the majority of cells. In these cells, co-localization was most often observed along the cell periphery, suggesting membrane-associated expression of the receptor (Figure 5J–L). During the remyelination phase, a further increase in APLNR signal intensity was observed, particularly in the CC-L. This increase was accompanied by a reduction in the number of NG2^+^ cells compared with the demyelination stage, although their numbers remained higher than in the control group. Notably, NG2^+^ cells in the CC-L displayed strong NG2 immunoreactivity with clear cytoplasmic localization and well-defined, homogeneous nuclei. Most NG2^+^ cells exhibited strong APLNR expression, with co-localization extending across most of the cell body and a prominent cytoplasmic distribution of the receptor (Figure 5M–O). In the CC-M, during remyelination, substantially fewer NG2^+^ cells were observed. These cells displayed an elongated or elliptical morphology, and both NG2 signal intensity and co-localization with APLNR were relatively weak, occurring only partially in occasional cells (Figure 5P–R). Colocalization analysis of NG2 and APLNR demonstrated distinct regional and group-dependent patterns across the CO, DE, and RE groups. In the CO group, the CC-L exhibited moderate Manders’ overlap (M2 ~0.7) but low Pearson’s correlation (~0.15), whereas the CC-M showed minimal spatial association, with Pearson’s values near zero and M2 values around 0.2, indicating weak or random distribution of the two markers. In the DE group, both the CC-L and CC-M displayed a modest increase in Pearson’s correlation to approximately 0.25, with comparable values between regions, suggesting a more uniform tissue response during demyelination. In the RE group, the strongest colocalization was observed, particularly in the CC-L, where Pearson’s correlation increased to approximately 0.4 and Manders’ coefficients reached ~0.85, consistent with pronounced spatial overlap and coordinated signal distribution. The CC-M in the RE group also showed increased colocalization compared with the corresponding control region, although values remained slightly lower than those in the CC-L (Figure 5S–U).

Quantitative colocalization analysis of APLNR and NG2 revealed a profound and statistically significant spatial reorganization during the RE phase compared to CO and DE. Assessment of signal correlation using Pearson’s *r* demonstrated a significant increase in the RE groups (RE-CC-L vs. CO-CC-L, *p* < 0.0001), indicating a shift from an independent spatial distribution in healthy tissue to a coordinated, positive linear relationship during remyelinization. Furthermore, co-occurrence measurements utilizing Manders’ coefficients highlighted a robust and specific spatial overlap driven by the remyelinating state. Manders’ M1 values were significantly elevated in the RE groups, establishing that the APLNR was expressed within NG2^+^ cells. Most notably, analysis of Manders’ M2 revealed a highly significant peak in the RE-CC-L group (*p* < 0.01 against all other groups), demonstrating that nearly the entire population of NG2^+^ cells became APLNR^+^—a characteristic largely absent in the CO group. Together, these statistical findings confirm a highly specific, coordinated upregulation and spatial alignment of APLNR on NG2^+^ cells that is unique to the remyelinization process. The complete results of the statistical analysis of Pearson’s r and Manders’ M1 and M2 coefficients are presented in Table 1. Overall, our results indicate a transition from low spatial association in control tissue to increased marker coordination during demyelination and pronounced colocalization during remyelination, with the most prominent effect observed in the CC-L.

### 2.4. Correlation Analysis

**Figure 6:**
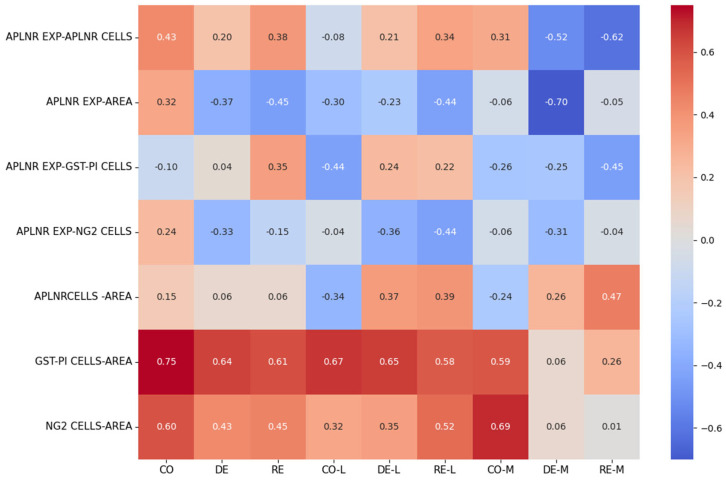
Heatmap of Pearson correlation coefficients. The heatmap displays Pearson’s correlation coefficients (r) for pairwise comparisons among APLNR expression intensity (APLNR EXP), APLNR^+^ cell density (APLNR CELLS), GST-π^+^ cell density (GST-PI CELLS), NG2^+^ cell density (NG2 CELLS), and corpus callosum area (AREA). Correlations were assessed across the whole corpus callosum (CO, DE, RE) as well as within its lateral (CO-L, DE-L, RE-L) and medial (CO-M, DE-M, RE-M) subregions. The color gradient indicates the direction and strength of the correlation, with red hues representing positive correlations and blue hues representing negative correlations. The exact r values are annotated within each respective cell. CO—control; DE—demyelination; RE—remyelination; L—lateral subregion; M—medial subregion.

To determine the contribution of cellular density to preservation or expansion of tissue volume, Pearson’s correlation analyses were performed between individual cell counts and area of CC in the control, demyelination and remyelination conditions. GST-π^+^ cells exhibited the strongest and most consistent positive correlation with CC area across all experimental phases. Significant positive associations were observed in controls (r = 0.75, *p* < 0.001), during demyelination (r = 0.64, *p* < 0.001) and during remyelination (r = 0.61, *p* < 0.001), supporting the interpretation that GST-π^+^ cells density is a principal determinant of CC volume. NG2^+^ OPCs also showed significant positive correlations with CC area in controls (r = 0.60, *p* < 0.001) and during remyelination (r = 0.45, *p* < 0.001). During demyelination the correlation remained significant but was weaker (r = 0.43, *p* < 0.001), consistent with an increase in OPC number that only partially tracks with preserved tissue area in the acute injury phase. By contrast, the absolute number of APLNR^+^ cells did not correlate significantly with CC area in either the control (*p* = 0.25) or demyelination (*p* = 0.63) groups. Additional marker-specific relationships were as follows: during remyelination APLNR expression correlated positively with GST-π^+^ cells density (r = 0.35, *p* = 0.030), whereas no significant APLNR–GST-π association was detected in controls (*p* = 0.53) or during demyelination (*p* = 0.81). Conversely, a significant negative correlation between APLNR expression and NG2^+^ cell number was observed specifically during demyelination (r = −0.33, *p* = 0.024); this relationship was not statistically significant in controls (*p* = 0.12) or during remyelination (*p* = 0.37). Together, these findings indicate that GST-π^+^ cells density is the dominant correlation of CC area, while APLNR shows phase-dependent associations with oligodendroglial markers—a positive link to GST-π^+^ cells during repair and an inverse relationship with OPC abundance during acute demyelination. Summary results of the correlation analysis are presented in Figure 6. The full results of the correlation analysis are presented in Table 2.

## 3. Discussion

The present study characterizes the temporal dynamics of APLNR in relation to NG2 and GST-π markers within the CC of C57BL/6 mice during cuprizone-induced demyelination and remyelination. Histological evaluation using LFB/Cresyl violet staining confirmed effective induction of demyelination, followed by partial structural recovery during the remyelination phase. Quantitative analyses demonstrated that demyelination was associated with a marked reduction in GST-π^+^ cells, accompanied by a pronounced increase in NG2^+^ oligodendrocyte precursor cells. During remyelination, GST-π^+^ cell density showed substantial recovery toward control levels, whereas NG2^+^ cell numbers declined but remained elevated compared with controls, consistent with established models of toxic demyelination [37]. In parallel, APLNR expression exhibited a progressive increase across experimental conditions, with low basal levels in controls, a significant elevation during demyelination, and a further increase during remyelination. This temporal pattern suggests that APLNR upregulation accompanies both the injury response and the subsequent repair phase. Although the present findings are primarily descriptive, the observed association between increased APLNR expression and oligodendroglial remodeling during remyelination raises the possibility that the apelin/APLNR signaling axis might contribute to regenerative processes in white matter. This potential role appears to extend beyond the previously reported involvement of this pathway in acute inflammatory responses [38].

### 3.1. Histomorphometric Verification of Demyelination and Remyelination in the CC

The efficacy of the cuprizone model was confirmed by qualitative and quantitative histological assessments of myelin in the CC using LFB/Cresyl violet staining. Our findings showed a clear reduction in CC area after 5 weeks of cuprizone exposure, followed by partial restoration after 5 weeks of remyelination. Although CC area is not yet widely established as a histomorphometric marker of demyelination, the present results are in line with previously reported ones [29,39]. They differ, however, from the observations of Toomey et al., who reported an increase in CC area after 3 weeks of cuprizone administration in a comparison of powdered and pellet cuprizone diets [4]. This discrepancy likely reflects differences in the duration of intoxication and the anatomical level analyzed, both of which are critical determinants of CC morphology. As noted by Toomey et al., changes in CC area during cuprizone exposure may predominantly reflect tissue edema rather than true hyperplasia or permanent tissue loss [4]. This interpretation is further supported by their finding of no difference in Hoechst^+^ cell density between groups, arguing against substantial cellular infiltration or proliferation as the main cause of the observed area increase [4]. Accordingly, CC area should be interpreted cautiously and in conjunction with myelin staining and cellular indices, particularly when distinguishing edema and acute inflammatory changes from genuine atrophy or recovery. Importantly, the histological changes reported by Toomey et al. were observed after 3 weeks of cuprizone administration, corresponding to an earlier phase of acute demyelination, when edema and inflammation are expected to predominate [4,11,21].

### 3.2. GST-π Dynamics in Demyelination and Remyelination

The present study demonstrates a marked, statistically significant loss of GST-π^+^ cells after 5 weeks of cuprizone exposure with partial repopulation following a 5-week remyelination period. Importantly, this effect was regionally heterogeneous: GST-π immunoreactivity in control animals was homogeneously distributed across the CC, with GST-π^+^ cells showing the compact, spheroidal cytoplasmic morphology characteristic of intact, functional oligodendrocytes. Following cuprizone supplementation, CC-L regions displayed focal deficits characterized by reduced numbers of spheroidal GST-π^+^ cells and markedly lower cytoplasmic staining, whereas CC-M regions primarily exhibited a diffuse decrease in staining intensity rather than complete cell loss. Thus, the medial oligodendrocyte population appears relatively more resistant, while lateral domains are particularly vulnerable to cuprizone-induced stress.

Quantitatively, GST-π cell density declined during DE and largely recovered during RE (approx. CO ≈ 2174, DE ≈ 1278, RE ≈ 2105 cells/mm^2^). However, recovery was not always complete at the subregional level, particularly in lateral compartments, suggesting that local reparative mechanisms (OPC mobilization, differentiation and metabolic recovery) are active but may be temporally constrained or incomplete. These spatially resolved findings emphasize that analysis of the whole CC can obscure biologically important local deficits. Therefore, lateral versus medial comparisons are critical when assessing cuprizone pathology.

Our correlation analyses further support a tight structure–cellularity relationship: across control, demyelinated and remyelinated groups the strongest and most consistent predictor of CC area was GST-π^+^ cell density (CO: r = 0.75; DE: r = 0.64; RE: r = 0.61). This observation indicates that the tissue atrophy seen in the cuprizone model largely reflects oligodendrocyte loss, whereas tissue restitution depends on successful OPC differentiation into mature myelinating cells. These results concord with our previously reported reduction in LFB intensity in CC [40], and with prior reports of regional susceptibility in the rostral CC [11,41,42].

Selective vulnerability to cuprizone likely reflects its action as a copper chelator and the downstream impairment of mitochondrial enzymes (cytochrome c oxidase, monoamine oxidase), producing mitochondrial dysfunction and energy failure—conditions that disproportionately affect metabolically active, myelinating cells and lead to reduced GST-π immunoreactivity [43]. Given the strong association between GST-π and mature oligodendrocytes, decreases in GST-π are commonly interpreted as oligodendrocyte loss [44]. Nonetheless, GST-π alone is not definitive. Several other markers (CC1, ASPA, NOGO-A) show overlapping but non-identical expression patterns, and changes in a single antigen may not faithfully reflect absolute cell numbers or functional competence [4,45].

During the recovery phase, the restoration of GST-π^+^ cell densities to near-control levels indicates successful spontaneous remyelination. Notably, our data revealed a more pronounced reduction in GST-π immunoreactivity within the lateral regions of the CC compared to the medial sector during demyelination, followed by a parallel recovery in both regions. These findings align with literature suggesting that the lateral portions of the rostral CC exhibit higher sensitivity to cuprizone and undergo more complete demyelination [11]. This recovery appears dependent on the differentiation of OPCs rather than the survival of damaged mature cells, mirroring the developmental transition where GST-π expression is initiated only post-differentiation [46]. Furthermore, the restoration of the spatial distribution of these cells suggests that repair mechanisms in the C57BL/6 CC are robust and capable of effectively repopulating the tissue architecture following toxin withdrawal [47].

Two important limitations arise from the biological properties of the markers. First, GST-π can be expressed in precursor populations and its intracellular localization matters: nuclear (N-type) localization has been associated with progenitors, whereas cytoplasmic (C-type) localization corresponds to mature oligodendrocytes [31]. Second, downregulation of a maturation marker under stress may mimic cell loss. Accordingly, combining GST-π with complementary mature oligodendrocyte markers (CC1, ASPA, PLP/MBP) and OPC markers (NG2, PDGFRα) is necessary to distinguish true cell loss from phenotypic modulation and to determine whether repopulating GST-π^+^ cells produce functional myelin [4].

### 3.3. NG2^+^ Cell Response and Proliferation

The present findings demonstrate a robust and dynamic response of NG2^+^ OPCs to cuprizone-induced demyelination and subsequent remyelination. In control animals, NG2^+^ cells were sparse and evenly distributed throughout the CC, whereas cuprizone exposure induced a marked increase in their number, accompanied by transient clustering and morphological alterations. Quantitatively, NG2^+^ cell density followed a CO < RE < DE pattern across the CC as well as in the CC-L and CC-M. This proliferative response is consistent with previously described OPC activation in demyelinated tissue and supports the concept that NG2^+^ cells represent an early component of the endogenous repair response following white matter injury [48,49,50,51].

A notable finding of the present study is the presence of regional heterogeneity in OPC dynamics. The CC-M appears to maintain a higher basal “reserve” of NG2^+^ cells under physiological conditions, whereas the lateral CC exhibits a greater relative increase during demyelination and, in some cases, slower or incomplete normalization during remyelination. Such spatial differences likely reflect variations in the local microenvironment, including vascularization, inflammatory activity, and the distribution of signaling molecules regulating OPC proliferation and differentiation [4,33,51]. Morphologically, NG2^+^ cells in demyelinated regions displayed thicker, shorter, and less branched processes, indicative of a reactive phenotype, which partially reverted to a more finely ramified morphology during remyelination. This morphological plasticity likely reflects transitions between activated, proliferative states and subsequent differentiation or restoration phases.

Correlation analysis further indicated that the relationship between NG2^+^ cell density and CC area is strongly influenced by both experimental stage and regional organization. In control animals, a pronounced positive correlation was observed, particularly in the medial region, consistent with the homeostatic maintenance of OPC populations relative to tissue volume [48,52]. During demyelination, CC area decreased markedly, especially in the lateral region, where histological evidence of demyelination was also most pronounced. Although NG2^+^ cell numbers increased substantially during this phase, confocal observations revealed nuclear pyknosis and disrupted NG2 proteoglycan labeling, suggesting that cuprizone toxicity may simultaneously stimulate OPC proliferation while inducing partial cell death. This dual effect may explain the pronounced demyelination observed in the CC-L regions and is consistent with previous reports describing regional vulnerability in the cuprizone model [4,11].

During the remyelination phase, CC area assumed intermediate values and a significant positive correlation with NG2^+^ cell density re-emerged, particularly in the lateral compartment. This finding suggests that regions initially most affected by demyelination become sites of intense OPC accumulation and proliferative activity during recovery. In contrast, the medial region exhibited a less dynamic or possibly temporally shifted response, further emphasizing the spatially heterogeneous nature of OPC regulation during white matter repair.

The observed OPC response likely reflects activation of signaling pathways associated with proliferation and differentiation. Evidence from previous studies indicates that a significant fraction of GPR17^+^/NG2^+^ cells can undergo terminal differentiation into GST-π^+^ mature oligodendrocytes during the early post-lesion phase, suggesting that at least part of the NG2^+^ population contributes directly to remyelination [49,50]. Regulation of OPC proliferation involves PDGF signaling and downstream intracellular pathways such as PI3K/mTOR and Wnt/β-catenin, which maintain the proliferative capacity of NG2^+^ cells [33]. However, persistent or dysregulated activation of Wnt/Tcf4 signaling may inhibit differentiation and delay remyelination, highlighting the importance of coordinated temporal regulation of signaling cascades rather than simple stimulation of OPC proliferation [33,53].

Another important consideration is the influence of genetic background and experimental models on OPC behavior. Comparative studies have demonstrated that NG2 responses differ between mouse strains, such as C57BL/6 and CD1, indicating that intrinsic molecular sensitivity to signaling pathways and differences in progenitor cell reserves can influence the magnitude and efficiency of OPC activation [22,33]. Furthermore, experiments involving Myrf mutations have shown that parenchymal OPCs constitute the primary source of early remyelination, whereas neural stem cells from the SVZ may contribute as a compensatory source when OPC differentiation is impaired. This illustrates the plasticity and functional interplay between distinct progenitor populations during myelin repair [54].

In a previous study we demonstrated significant depletion of NG2^+^ cells in the SVZ during demyelination, which was interpreted as migration toward the primary site of injury in the CC [29]. Such SVZ-to-CC migration further emphasizes the complex spatial organization of progenitor responses and suggests that multiple neurogenic niches may contribute to repair mechanisms in demyelinating conditions [54].

Interpretation of quantitative changes in NG2 density should also be considered alongside the dynamics of other oligodendrocyte lineage markers. The reduction in GST-π^+^ cells during demyelination and their partial recovery during remyelination support the hypothesis that a subset of NG2^+^ OPCs differentiates into mature oligodendrocytes during the repair process. However, restoration of GST-π expression alone does not necessarily indicate complete recovery of functional myelin sheaths [4,45]. Moreover, the intracellular localization of GST-π, nuclear (N-type) versus cytoplasmic (C-type), together with co-expression patterns with precursor and mature markers provides additional information about lineage progression and should be considered when interpreting OPC activation and differentiation [31].

### 3.4. The Role of APLNR in Demyelination and Remyelination

The apelinergic system has been implicated in the regulation of CNS homeostasis and pathology, exerting both anti-inflammatory and, under certain conditions, pro-inflammatory effects through modulation of microglial cytokine responses [55,56,57]. Some authors describe it as a modulator and suppressor of neuroinflammation, reducing the immune cells infiltration in the brain and having positive effect towards the severity of experimental autoimmune encephalitis and experimental rat models of Alzheimer’s disease [55,56]. Conversely, other articles showcase its pro-inflammatory role by promoting higher expression levels of cytokines in microglial cells [38]. The correlation analysis between APLNR expression and the number of GST-π^+^ cells in the CC revealed a complex and region-dependent relationship between the two markers. When the whole coronal section of the rostral CC was analyzed, no significant correlation was observed in either the control group or during the demyelination phase. In contrast, during remyelination a positive and statistically significant correlation between APLNR expression and the number of GST-π^+^ cells was detected, suggesting a potential supportive role of APLNR in the restoration of GST-π^+^ cells.

Subgroup analysis provided a more detailed spatial pattern. In the control group, both the CC-L and CC-M showed a negative association between APLNR and GST-π expression, although statistical significance was reached only in the CC-L. During both demyelination and remyelination, a positive correlation between APLNR and GST-π was predominantly observed in the CC-L, which represent the areas most strongly affected by demyelination, evident on the LFB-stained sections. In contrast, the medial regions consistently demonstrated a negative correlation across all experimental groups, with the negative correlation reaching statistical significance in the medial region during remyelination. Taken together, these observations indicate a complex, bidirectional relationship between APLNR expression and the population of GST-π^+^ cells. APLNR may be associated with mechanisms that support remyelination in the more severely affected CC-L, while participating in distinct regulatory processes in the relatively less affected CC-M [39]. In this context, our findings support the key role of APLNR in myelination previously reported by Ito et al. [58], while additionally providing evidence for a spatially and phase-dependent relationship between APLNR expression and the number of GST-π^+^ cells in the CC.

Moreover, in the present study, we observed clear co-localization of APLNR with NG2^+^ cells during demyelination that became most pronounced during the remyelination phase. These findings suggest that APLNR may participate in the activation and/or differentiation of OPCs and are supported statistically by Pearson’s r value and Mandler’s M1 and M2 coefficients assessment. This observation complements our previous reports demonstrating markedly increased APLNR expression in the SVZ during demyelination with subsequent normalization during remyelination [29]. Notably, the pattern observed here in the CC differs from the canonical behavior of many inflammatory markers, which typically peak during the acute injury phase and decline during tissue repair [17]. Instead, the remyelination-associated increase in APLNR suggests a possible role for apelin signaling in the maturation or maintenance of newly generated cells. This interpretation is supported by evidence that apelin promotes neural stem cell maturation, suppresses apoptosis, and reduces oxidative stress [57]. In the context of cuprizone intoxication, which induces mitochondrial dysfunction and oxidative injury, APLNR upregulation may therefore reflect an endogenous protective response aimed at limiting toxic damage and supporting repair [59]. It is also important to emphasize that our previous study [29] focused on the SVZ, a distinct neurogenic niche, whereas the present work examined the CC, a separate white matter region. Accordingly, the current findings do not contradict our earlier observations; rather, they complement them by showing that APLNR regulation is region-specific and is accompanied in the CC by dynamic changes in NG2^+^ cells during demyelination and remyelination.

Functionally, the increased APLNR expression observed in the CC, which peaked during remyelination, contrasts with typical inflammatory patterns and therefore points toward a potential role for apelinergic signaling in supporting the survival or maturation of newly generated cells rather than merely reflecting inflammatory activity. The protective, anti-apoptotic, and anti-oxidative effects of apelin signaling described in previous studies provide a plausible mechanism by which increased APLNR expression may counteract cuprizone-induced mitochondrial oxidative stress [57,59].

The spatial pattern identified in the present study, namely the accumulation of NG2^+^ cells within demyelinated regions of the CC accompanied by a reduction in progenitors in the SVZ, is consistent with the migration of progenitor cells toward sites of injury. In this context, apelin/APLNR signaling could act as a chemotactic and pro-survival signal facilitating this process. Stromal and localized signaling cues that guide progenitor migration and survival have been well documented, and apelin signaling has been shown to enhance cell motility and survival in inflammatory microenvironments [34,48,60,61]. Moreover, apelin has been reported to exert immunomodulatory effects that attenuate immune cell infiltration and neuroinflammation, potentially creating a microenvironment more permissive for the survival and integration of SVZ-derived progenitors [23].

Our correlation analysis revealed a spatially restricted moderate inverse relationship between APLNR expression and NG2^+^ cell counts. When analyzing the whole coronal section of the rostral CC of demyelinated animals, as well as in the CC-L of both the demyelination and remyelination groups, higher APLNR levels were associated with lower NG2 expression, whereas this relationship was not observed in the medial regions. Two non-exclusive mechanisms may explain this apparent discrepancy between cellular co-localization and negative correlation. First, activation of APLNR signaling may restrain OPC proliferation, leading to lower NG2 expression in areas where receptor levels are elevated. Alternatively, and perhaps more plausibly given the concurrent increase in GST-π^+^ cells observed in these regions, APLNR signaling may promote OPC differentiation into mature oligodendrocytes, thereby reducing NG2 expression as progenitors transition toward a differentiated phenotype. Supporting this interpretation, previous studies have shown that modulation of APLNR signaling can influence myelination processes, with impaired myelination observed in APLNR-deficient animals and enhanced myelin formation following receptor activation [58].

The colocalization analysis demonstrated a clear stage- and region-dependent reorganization of the relationship between APLNR and NG2^+^ cells across control, demyelination, and remyelination conditions. In the control group, the CC-L exhibited moderate Manders’ overlap (particularly M2), but very low Pearson’s correlation, indicating that although APLNR and NG2 signals frequently occupied the same spatial domains, their intensity distributions were not coordinated. This pattern is consistent with spatial proximity without functional coupling. In contrast, the CC-M control region showed minimal interaction, with near-zero Pearson’s coefficients and very low Manders’ values, suggesting largely independent and stochastic distribution of both markers under physiological conditions. During demyelination, both CC-L and CC-M regions displayed an increase in Pearson’s correlation compared to control, indicating the emergence of a more coordinated relationship between APLNR and NG2 expression. However, Manders’ coefficients remained comparatively low, suggesting that while the two signals began to covary in intensity, substantial spatial co-accumulation had not yet occurred. Importantly, the distinction between CC-L and CC-M regions became less pronounced in this phase, implying that demyelination induces a more homogeneous, tissue-wide reorganization of cellular signaling patterns rather than region-restricted effects. The most pronounced colocalization was observed during remyelination, particularly in the CC-L, where both Pearson’s r and Manders’ coefficients (M1 and M2) reached their highest values. This combination of strong intensity correlation and high reciprocal overlap is consistent with true colocalization, indicating that APLNR and NG2 are not only spatially co-distributed but also tightly coupled in their expression dynamics. This suggests their participation in a shared cellular or subcellular compartment during the remyelination process, potentially reflecting coordinated signaling within NG2^+^ oligodendrocyte precursor cells. Although the CC-M remyelination region also demonstrated a substantial increase relative to both control and demyelination, it remained slightly lower than the CC-L region, indicating regional heterogeneity in the efficiency or extent of reparative responses. Taken together, the data describe a progressive transition from spatial independence under physiological conditions to partial intensity coordination during demyelination, culminating in robust structural and functional colocalization during remyelination. The most striking change was observed in the CC-M region, where Manders’ M2 increased markedly from a low baseline in control to substantially higher values during remyelination, consistent with a recruitment process in which a previously APLNR-poor NG2^+^ population becomes increasingly APLNR-positive during repair. Overall, the colocalization of APLNR with NG2^+^ cells during remyelination, supported by Pearson’s r and Manders’ M1/M2 coefficients, together with the spatially selective negative correlation between receptor expression and NG2 density in the most affected CC-L regions of the CC, suggests that apelinergic signaling may facilitate the transition of NG2^+^ OPCs from a reactive or proliferative state toward terminal differentiation and survival. This interpretation aligns with prior evidence that NG2^+^ progenitors proliferate following demyelinating injury but may remain arrested in an immature state, and that overcoming this differentiation block is critical for successful remyelination [49,50,51].

Molecular characterization of NG2^+^ cells reveals significant regional distinctions between those residing in the SVZ and the CC parenchyma. In the SVZ, NG2^+^ cells have been described as “type C-like” multipotent progenitors that express markers of neural stem cells such as the Lewis X (LeX) antigen, epidermal growth factor receptor (EGFR), and transcription factors Dlx2 and Mash1. Furthermore, a subset of SVZ NG2^+^ cells displays immature neuronal markers like PSA-NCAM and class III β-tubulin, reflecting a molecular profile capable of generating hippocampal GABAergic interneurons. Conversely, NG2^+^ cells in the CC and broader parenchyma are predominantly restricted to the oligodendrocyte lineage. While they share the expression of platelet-derived growth factor receptor-α (PDGFRα) with SVZ NG2^+^ cells, they are reported to lack the neurogenic markers found in the SVZ niche. Proliferative dynamics also differ sharply, as NG2^+^ cells constitute over 94% of the actively cycling population in the parenchyma but represent less than 1% of the proliferating cells within the SVZ, where other progenitor types predominate. While some studies suggest these populations may be antigenically indistinguishable in certain contexts, the potential for SVZ-derived NG2^+^ cells to express neurogenic molecular cues remains a key distinction from the strictly oligodendroglial parenchymal population [62,63]. While our previous work demonstrated that NG2^+^ cell density in the SVZ significantly decreases during demyelination [29], the current data show a robust, reactive proliferation of NG2^+^ progenitors in the CC that increases significantly during both the demyelination and remyelination phases. Most importantly, this paper provides novel evidence regarding APLNR dynamics; in contrast to the low NG2/APLNR co-localization observed in the SVZ, we report a significant upregulation and enhanced spatial coupling of APLNR within the NG2^+^ population specifically in the lateral CC during remyelination. This high spatial co-localization, characterized by an M2 coefficient of approximately 0.85, suggests that APLNR signaling in the CC is uniquely positioned to facilitate the terminal differentiation of progenitors at the primary site of myelin damage. 

Nevertheless, the present study provides primarily a descriptive, morphological characterization of the spatial and temporal changes in APLNR, GST-π, and NG2 expression during demyelination and remyelination in the rostral CC, together with correlation analyses. As such, the findings do not establish causal relationships, and further mechanistic studies are required to determine the functional significance of these associations. Thus, extrapolation of these findings to human demyelinating diseases requires caution. The cuprizone model preserves the intrinsic differentiation capacity of OPCs, whereas conditions such as MS involve complex autoimmune and chronic inflammatory components that may limit OPC maturation. Consequently, therapeutic strategies aimed solely at increasing OPC numbers may be insufficient unless combined with approaches that promote differentiation and address the underlying inflammatory microenvironment [53,60,64,65,66].

### 3.5. Limitations

The present study provides detailed histological and quantitative data on APLNR, NG2^+^ OPCs and GST-π^+^ oligodendrocytes in the cuprizone model. However, several limitations should be considered when interpreting the findings:

1. Model-specific limitations

The cuprizone model isolates toxic demyelination from adaptive immune responses and therefore does not reproduce the autoimmune-inflammatory milieu of MS. This limits direct extrapolation of APLNR-related mechanisms to immune-mediated human disease [5]. Strain dependency is well documented: results obtained in C57BL/6 mice (used here) may differ in other strains that show different sensitivity and temporal profiles of demyelination/remyelination (CD1). Thus, generalizability across models and genotypes is limited [11].

2. Correlative (non-causal) evidence

Our data are observational and rely on spatial and quantitative correlations between APLNR and lineage markers. Without targeted loss- or gain-of-function experiments (cell-type specific APLNR manipulation, receptor agonists/antagonists, or lineage tracing), causality cannot be established. Prior work showing APLNR effects on myelination is supportive but not definitive for the mechanisms described here [58].

3. Reliance on immunohistochemistry and marker interpretation

Immunohistochemical readouts depend on antibody specificity, epitope accessibility, and subcellular localization (e.g., GST-π N- vs. C-type). Changes in marker intensity can reflect altered expression, translocation, or true cell loss; single-marker interpretation may therefore be misleading. Co-labeling with multiple mature and progenitor markers partially mitigates this, but additional validation (e.g., CC1, ASPA, PLP/MBP, PDGFRα, and molecular assays) would strengthen conclusions.

Quantification was performed on 2D histological sections and ROI selection may not fully capture 3D tissue heterogeneity; stereological approaches or volumetric imaging would provide more robust estimates of absolute cell numbers.

4. Temporal and sampling constraints

We sampled defined time points (5-week demyelination; 5-week remyelination). Important early or late dynamics (very early OPC activation, transient APLNR peaks, or long-term durability of remyelination) may have been missed. More frequent sampling would clarify temporal relationships and windows of opportunity for intervention.

5. Functional readouts missing

The study links cellular markers to tissue area but lacks ultrastructural (electron microscopy) confirmation of compact myelin and electrophysiological measures of axonal conduction. Restoration of marker-positive oligodendrocytes does not guarantee formation of functional myelin sheaths; future studies should integrate EM and functional testing to corroborate structural repair [46,47].

6. Potential technical confounders

Cuprizone dosing via drinking water may introduce variability in individual intake and effective exposure; although we homogenized preparations, per-animal consumption differences can affect lesion severity.

Imaging and quantification were performed with fixed microscope settings to reduce bias, but observer blinding, automated segmentation, and inter-rater reliability metrics would further minimize subjective influence.

7. Statistical power and multiple comparisons

Some subgroup analyses (regional correlations, pairwise comparisons) involved modest sample sizes and multiple testing; although nonparametric tests and corrections were applied, there remains a risk of type I/II errors in smaller comparisons. Larger cohorts would improve robustness.

8. Translational caveats

The cuprizone model preserves intrinsic OPC differentiation potential, unlike chronic, immune-mediated MS lesions where differentiation is often blocked. Thus, therapeutic strategies suggested by our data (e.g., modulation of APLNR to promote OPC differentiation) will need testing in immune-competent and chronic lesion models before clinical translation [53,60].

## 4. Materials and Methods

### 4.1. Experimental Animals

The experiment was conducted on 8-week-old male mice of the C57BL/6 strain from the vivarium of the Medical Faculty at the Medical University of Sofia. The animals were maintained under standard housing conditions (12:12 h light/dark cycle, temperature 22 ± 2 °C, free access to food and water) in accordance with the ethical requirements for work with laboratory animals. All procedures were approved by the Bulgarian Food Safety Agency (BFSA) under permit No. 416/19 December 2024.

### 4.2. Cuprizone Model

The demyelination model was induced by administration of the neurotoxic agent cuprizone (chemical: bis(cyclohexanone-oxalyl-dihydrazone) CAS: 370-81-0, Sigma Aldrich, Vienna, Austria) in 8-week-old C57BL/6 mice, according to described protocols [21,29]. Cuprizone was administered via the drinking water at a concentration of 0.2% (*w*/*v*). The mixture was homogenized with a magnetic stirrer for approximately 30 min and resuspended several times a day to ensure even distribution. The mixture was changed daily.

### 4.3. Experimental Groups

1. Control group—received plain drinking water.
2. Demyelination group—cuprizone 0.2% in drinking water for 5 weeks.
3. Remyelination group—cuprizone 0.2% for 5 weeks, followed by a 5-week period without cuprizone (remyelination).

### 4.4. Tissue Preparation

The animals were deeply anesthetized by intraperitoneal injection of sodium thiopental (Sigma Aldrich) at a dose of 30 mg/kg. After full immobilization, transcardial perfusion was carried out via a cannula inserted into the left ventricle and secured in the ascending aorta. Initially, the circulatory system was flushed with 0.05 M phosphate-buffered saline (PBS) (Sigma Aldrich; cat. No. 1465920006), pH 7.4 (approximately 5 min), followed by perfusion with chilled 4% paraformaldehyde (Sigma Aldrich; cat. No. 30525-89-4) dissolved in 0.1 M PBS for ~20 min. The head was removed and a craniotomy was subsequently performed; the brain was carefully extracted and placed for postfixation in the same paraformaldehyde solution at 4 °C for 12–18 h. The next day the brain was cut coronally through the midbrain (between the superior and inferior colliculi) and both hemispheres were embedded in paraffin for subsequent analyses.

### 4.5. Histological Assessment with LFB/Cresyl Violet

Serial coronal sections (6 µm thick) were cut from paraffin-embedded tissue blocks using a microtome (Leica RM2155, Nussloch, Germany). Sections selected for analysis were obtained from the rostrocaudal interval between bregma +1.10 and +0.02 mm, according to the Paxinos and Franklin mouse brain atlas [67]. The sections were mounted on adhesive slides, deparaffinized, and rehydrated through graded ethanol to 95% ethanol. Myelin staining was then performed with 0.01% Luxol Fast Blue (LFB; Sigma-Aldrich, St. Louis, MI, USA, cat. no. L0294) at 58 °C for 6 h. Differentiation was carried out in 0.05% lithium carbonate solution (Sigma-Aldrich, cat. no. 255823), followed by counterstaining with Cresyl violet (Sigma-Aldrich, cat. no. 41830-80-2). After air-drying, the sections were coverslipped with Entellan (Merck, Darmstadt, Germany, cat. no. 1079600500). For microscopic analysis, every fifth coronal section within this interval was documented at 100× magnification.

### 4.6. Immunohistochemistry

Immunohistochemistry was performed for the purposes of the present study following a standard protocol [29].

Sections (6 µm thick) were deparaffinized and rehydrated by three-step processing with three changes in xylene (Merck; cat. No. 108298400) (III–I), 5 min each, followed by a descending alcohol series: 100%, 95%, 90%, 80%, 70% ethanol (Merck; cat. No. 1009835000) and distilled water (twice, 3 min each). Antigen retrieval was performed in 10 mM citrate buffer (ScyTek Laboratories Inc., Logan, UT, USA; cat. No. CPL500) at pH 6.0 by boiling in a water bath at 95 °C for 20 min. After cooling, sections were washed three times for 5 min each in TTBS (Elabscience, Wuhan, China; E-BC-R335). Endogenous peroxidase was blocked with 3% H_2_O_2_ in distilled water for 10 min, followed by three washes of 5 min each in TTBS. Sections were then incubated with Superblock (ScyTek Laboratories Inc.) for 5 min at room temperature. Endogenous biotin was blocked using the corresponding kit (ScyTek Laboratories Inc.; cat. No. BBK120) according to the kit instructions: 15 min with part A, wash, then 15 min with part B, followed by three washes in TTBS. To prevent nonspecific binding of biotinylated anti-mouse immunoglobulins to endogenous mouse immunoglobulins, sections were treated with a mouse-to-mouse blocking kit (ScyTek Laboratories Inc.; cat. No. MTM015) for 1 h at room temperature, followed by three washes in TTBS. After three 5 min washes in TTBS, sections were incubated with the pre-diluted primary antibodies—polyclonal anti-APLNR (E-AB-13919, Elabscience Biotechnology^®^) in 1:50 dilution, monoclonal anti-NG2 (SC-53389, Santa Cruz Biotechnology Inc., Dallas, TX, USA) in 1:50 dilution, polyclonal anti-GST-π (ADI-MSA-102-E, Enzo Life Sciences, Farmingdale, NY, USA)—for 18 h at 4 °C. This was followed by incubation with a biotinylated anti-rabbit/anti-mouse secondary antibody for 10 min at room temperature. After three 5 min washes in TTBS, sections were incubated with HRP (ScyTek Laboratories Inc.; cat. No. AFN600) for 10 min and, following another three 5 min washes in TTBS, the reaction was developed with the chromogen 3,3′-diaminobenzidine (DAB; Sigma Aldrich) twice for 5 min under visual control in the dark. The reaction was stopped with distilled water, after which sections were counterstained with Mayer’s hematoxylin for 5 min and differentiated in tap water for 10 min. After dehydration and clearing in xylene (2 × 10 min), the sections were coverslipped with Entelan (Merck; cat. No. 1079600500).

### 4.7. Immunofluorescence

Sections were deparaffinized in three changes in xylene (Merck; cat. No. 1082984000) for 10 min each and rehydrated through a descending ethanol series (100%, 95%, 90%, 80%, 70%; Merck; cat. No. 1009835000) followed by distilled water, 5 min per step. Antigen retrieval was performed in 10 mM citrate buffer (ScyTek Laboratories Inc.; cat. No. CPL500) at pH 6.0 by heating in a water bath at 95 °C for 20 min. After cooling, sections were washed three times for 5 min in TTBS (Elabscience; E-BC-R335) and incubated with Superblock (ScyTek Laboratories Inc.) for 5 min at room temperature.

To prevent nonspecific binding of anti-mouse immunoglobulins to endogenous mouse immunoglobulins, sections were treated with a mouse-to-mouse blocking kit (ScyTek Laboratories Inc.; cat. No. MTM015) for 1 h at room temperature, followed by three washes in TTBS. The sections were then incubated with primary antibodies—polyclonal anti-APLNR (Elabscience; E-AB-13919), monoclonal anti-NG2 (Santa Cruz Biotechnology Inc.; sc-53389), and polyclonal anti-GST-π (ADI-MSA-102-E, Enzo Life Sciences, Farmingdale, NY, USA)—for 18 h at 4 °C.

Following primary antibody incubation, sections were incubated for 1 h at room temperature with secondary antibodies: anti-rabbit IgG (Elab Fluor^®^ 488; Elabscience; E-AB-1055) and anti-mouse IgG (Elab Fluor^®^ 594; Elabscience; E-AB-1059). After three washes in TTBS (5 min each), nuclear counterstaining was performed with Hoechst 33342 (Santa Cruz Biotechnology Inc.; sc-391054) for 10 min. Sections were then washed three times in TBS and mounted with FluoreGuard Mounting Medium (Hard Set) (ScyTek Laboratories Inc.; FMH030), coverslipped, and stored in the dark until imaging.

### 4.8. Digital Imaging Acquisition and Analysis

All immunohistochemistry-stained sections were examined and documented using an upright microscope system, Olympus BX83 (Tokyo, Japan), interfaced with a computer. Microscope configuration (light source, filter sets and camera settings) was calibrated and maintained constant for all image acquisitions; brightness, contrast and color balance were not altered between samples. Brightfield images were acquired at 100×, 400× and 600× total magnification (objectives 10×, 40× and 60×) and exported as TIFF files. A scale bar was added to each image using the acquisition software and its accuracy was verified with a stage micrometer. Immunofluorescent specimens were imaged on a confocal system, Leica TCS SPE, and digital images were exported as TIFF files; scale bars were applied and verified as above.

Quantitative image analysis was performed using the Java-based platform ImageJ (NIH; Version: 1.54s) following a standardized workflow. Images were converted to 8-bit grayscale (0–255), and regions of interest (ROIs) encompassing only the CC were delineated. Mean gray value within each ROI was measured and values were inverted so that stronger staining corresponded to higher numerical values (0 = white; 255 = black). CC area was measured with the freehand selection and ImageJ area tool and reported in mm^2^ after calibration to the image scale bar automatically generated during acquisition. Immunopositive cells within the CC were counted in fields captured at 400×, field area was measured as above, and cell counts were normalized to cells per mm^2^ in accordance with established protocols [4,11,68,69]. To quantify the spatial relationship between APLNR and NG2, double-labeled fluorescence micrographs were analyzed using ImageJ software. Colocalization was assessed by calculating Pearson’s correlation coefficient (r), which evaluates the linear correlation between signal intensities in the two channels. In addition, Manders’ overlap coefficients were calculated to determine the proportion of co-occurring signals. Specifically, M1 represented the fraction of the total APLNR signal overlapping with NG2, whereas M2 represented the fraction of the total NG2 signal overlapping with APLNR. The analysis was conducted following a standard protocol [70]. For detailed morphological analysis, the CC was divided along the midline into left and right hemispheric halves, and each half was further subdivided into CC-M and CC-L compartments according to the anatomical boundaries described by Kipp [11] and applied in our previous study [40].

### 4.9. Statistical Analysis

Statistical analyses were performed using GraphPad Prism 10.1.1 (GraphPad Software). Data were first assessed for normality with the Shapiro–Wilk test. Variables that deviated from a Gaussian distribution were compared between groups using the Kruskal–Wallis test followed by Dunn’s multiple-comparison post hoc test. Comparisons between CC-L and CC-M were made with the Mann–Whitney U test. Associations between variables were evaluated using Spearman’s rank correlation. Data are reported as mean ± standard deviation or median (interquartile range), as appropriate, and statistical significance was set at *p* < 0.05.

## 5. Conclusions

The present study demonstrates that APLNR expression undergoes marked and spatially heterogeneous changes in the CC during cuprizone-induced demyelination and subsequent remyelination. The progressive association of APLNR immunoreactivity with NG2^+^ OPCs, together with the decline in GST-π^+^ cells during demyelination and their partial recovery during remyelination, indicates that APLNR may be linked to the endogenous cellular response to myelin loss and restoration. These findings are further supported by the corresponding structural changes in the CC, which mirror the temporal pattern of demyelination and recovery. Although the present data are descriptive and do not establish causality, they suggest that the apelin/APLNR axis may participate in oligodendrocyte lineage dynamics during myelin injury and repair. Further functional studies are needed to define the mechanistic role of APLNR signaling and to assess its potential as a therapeutic target in demyelinating disease.

## Figures and Tables

**Table 1:** Statistical Analysis of APLNR and NG2 Colocalization Across Experimental Groups.

Comparison	Pearson’s r (*p*-Value)	Manders’ M1 (*p*-Value)	Manders’ M2 (*p*-Value)
CO-CC-L vs. CO-CC-M	<0.0001	0.0659	<0.0001
CO-CC-L vs. DE-CC-L	0.0169	0.8743	<0.0001
CO-CC-L vs. DE-CC-M	0.0199	0.0428	<0.0001
CO-CC-L vs. RE-CC-L	<0.0001	<0.0001	0.0042
CO-CC-L vs. RE-CC-M	0.0013	<0.0001	0.9289
CO-CC-M vs. DE-CC-L	<0.0001	0.5187	0.0014
CO-CC-M vs. DE-CC-M	<0.0001	1.0000	<0.0001
CO-CC-M vs. RE-CC-L	<0.0001	<0.0001	<0.0001
CO-CC-M vs. RE-CC-M	<0.0001	0.0001	<0.0001
DE-CC-L vs. DE-CC-M	1.0000	0.4105	0.0015
DE-CC-L vs. RE-CC-L	0.0006	<0.0001	<0.0001
DE-CC-L vs. RE-CC-M	0.9584	<0.0001	<0.0001
DE-CC-M vs. RE-CC-L	0.0005	<0.0001	<0.0001
DE-CC-M vs. RE-CC-M	0.9451	0.0002	0.0010
RE-CC-L vs. RE-CC-M	0.0087	0.6322	0.0002

**Table 2:** Results of the correlation analysis.

Dataset	Group	R (Correlation)	*p*-Value
**APLNR EXP/AREA**	CO	0.3224	0.0328
	DE	−0.3703	0.0104
	RE	−0.4477	0.0048
	CO-L	−0.3040	0.1311
	DE-L	−0.2259	0.1920
	RE-L	−0.4374	0.0177
	CO-M	−0.0575	0.8209
	DE-M	−0.7040	0.0106
	RE-M	−0.0496	0.8784
**APLNR EXP/GST-π CELLS**	CO	−0.0968	0.5320
	DE	0.0363	0.8086
	RE	0.3517	0.0304
	CO-L	−0.4422	0.0237
	DE-L	0.2431	0.1593
	RE-L	0.2172	0.2578
	CO-M	−0.2605	0.2965
	DE-M	−0.2503	0.4327
	RE-M	−0.4460	0.1462
**APLNR EXP/NG2 CELLS**	CO	0.2391	0.1181
	DE	−0.3279	0.0244
	RE	−0.1494	0.3706
	CO-L	−0.0381	0.8536
	DE-L	−0.3646	0.0313
	RE-L	−0.4351	0.0183
	CO-M	−0.0572	0.8218
	DE-M	−0.3055	0.3343
	RE-M	−0.0384	0.9058
**APLNR CELLS/AREA**	CO	0.1528	0.2480
	DE	0.0632	0.6256
	RE	0.0605	0.6700
	CO-L	−0.3364	0.0418
	DE-L	0.3658	0.0124
	RE-L	0.3897	0.0188
	CO-M	−0.2393	0.2834
	DE-M	0.2609	0.3291
	RE-M	0.4654	0.0693
**GST-π CELL/AREA**	CO	0.7544	<0.0001
	DE	0.6376	<0.0001
	RE	0.6116	<0.0001
	CO-L	0.6673	<0.0001
	DE-L	0.6466	<0.0001
	RE-L	0.5842	<0.0001
	CO-M	0.5918	<0.0001
	DE-M	0.0571	0.7063
	RE-M	0.2650	0.0602
**NG2 CELLS/AREA**	CO	0.6001	<0.0001
	DE	0.4322	0.0001
	RE	0.4476	0.0002
	CO-L	0.3220	0.0273
	DE-L	0.3477	0.0155
	RE-L	0.5156	0.0006
	CO-M	0.6890	0.0001
	DE-M	0.0626	0.7663
	RE-M	0.0078	0.9706

## Data Availability

The original contributions presented in this study are included in the article. Further inquiries can be directed to the corresponding author.

## Abbreviations

APLNR	Apelin receptor
AREA	Corpus callosum area (morphometric measurement)
BFSA	Bulgarian Food Safety Agency
CC	Corpus callosum
CC-L	Lateral corpus callosum
CC-M	Medial corpus callosum
CNS	Central nervous system
CO	Control group
CPZ	Cuprizone
DE	Demyelination group
DE-L	Lateral demyelination group
DE-M	Medial demyelination group
DAB	3,3′-Diaminobenzidine
EAE	Experimental autoimmune encephalomyelitis
GST	Glutathione S-transferase
GST-π	Glutathione S-transferase pi isoform
HRP	Horseradish peroxidase
LPC	Lysophosphatidylcholine (lysolecithin)
MOG	Myelin oligodendrocyte glycoprotein
MS	Multiple sclerosis
NG2	Neural/glial antigen 2 (CSPG4 proteoglycan)
OPC	Oligodendrocyte progenitor
